# The multifaceted role of kallistatin in human diseases: mechanistic insights and translational potential

**DOI:** 10.3389/fcvm.2025.1701235

**Published:** 2026-01-05

**Authors:** Minrong Yu, Yanqing Feng, Zhiyan Wu, Suchun Li

**Affiliations:** 1 Department of Nephrology, The First Affiliated Hospital of Sun Yat-Sen University, Guangzhou, China; 2 NHC Key Laboratory of Clinical Nephrology and Guangdong Provincial Key Laboratory of Nephrology, Sun Yat-Sen University, Guangzhou, China; 3 Zhongshan School of Medicine, Sun Yat-Sen University, Guangzhou, China; 4 Guanghua School of Stomatology, Sun Yat-Sen University, Guangzhou, China

**Keywords:** biomarker, kallistatin, organ injury, signaling cascades, therapeutics

## Abstract

Kallistatin, a multifunctional serine protease inhibitor, is widely distributed with tissue-specific effects. It may serve as a new diagnostic biomarker and therapeutic target for human diseases. Through binding to its two structural elements and specific receptors, it regulates differential signaling cascades, and thus has a wide spectrum of biological functions. In cardiovascular diseases like hypertension, atherosclerosis, and heart failure, it exerts protective effects by improving endothelial function, anti-inflammation, and regulating lipid metabolism. In liver diseases, high hepatic expression correlates with nonalcoholic fatty liver disease, while decreased serum levels indicate severe cirrhosis or liver fibrosis. In metabolic diseases, it regulates insulin resistance, glucose metabolism, angiogenesis and inflammation. In inflammatory diseases, its role is dual: it attenuates inflammation in rheumatoid arthritis, sepsis, etc., but exacerbates chronic rhinosinusitis and autoimmune uveitis by promoting inflammatory cytokines secretion. In cancer, it inhibits tumor cell proliferation, angiogenesis, and metastasis, with lower tumor tissue expression linked to cancer development. Kallistatin also serves as a potential biomarker for chronic kidney disease, preterm birth, neurodegenerative diseases, and other diseases. This review synthesizes current knowledge on kallistatin's mechanisms in organ injury and repair, emphasizes its therapeutic potential across disease contexts, and discusses challenges and future directions for clinical translation, including organ-targeted strategies and combination therapies.

## 1 Introduction

Kallistatin, also known as serpin family A member 4 (SERPINA4), is a member of the serine proteinase inhibitors (SERPIN) superfamily. It was first discovered in human plasma as a kallikrein-binding protein (KBP) that effectively inhibited the activity of tissue kallikrein and plasma kallikrein (1–3). Kallistatin is widely distributed in various organs and body fluids, especially those related to cardiovascular function. The physiological concentration of kallistatin in human plasma is approximately 22.1 ± 3.5 μg/ml (4). Notably, the highest kallistatin concentration was observed in the kidney, followed by the liver, lung, prostate gland, and aorta, while the pancreas and brain exhibited a relatively lower concentration of kallistatin (5). This distinct expression pattern suggests its fundamental role in maintaining multi-organ homeostasis.

**Figure 1:**
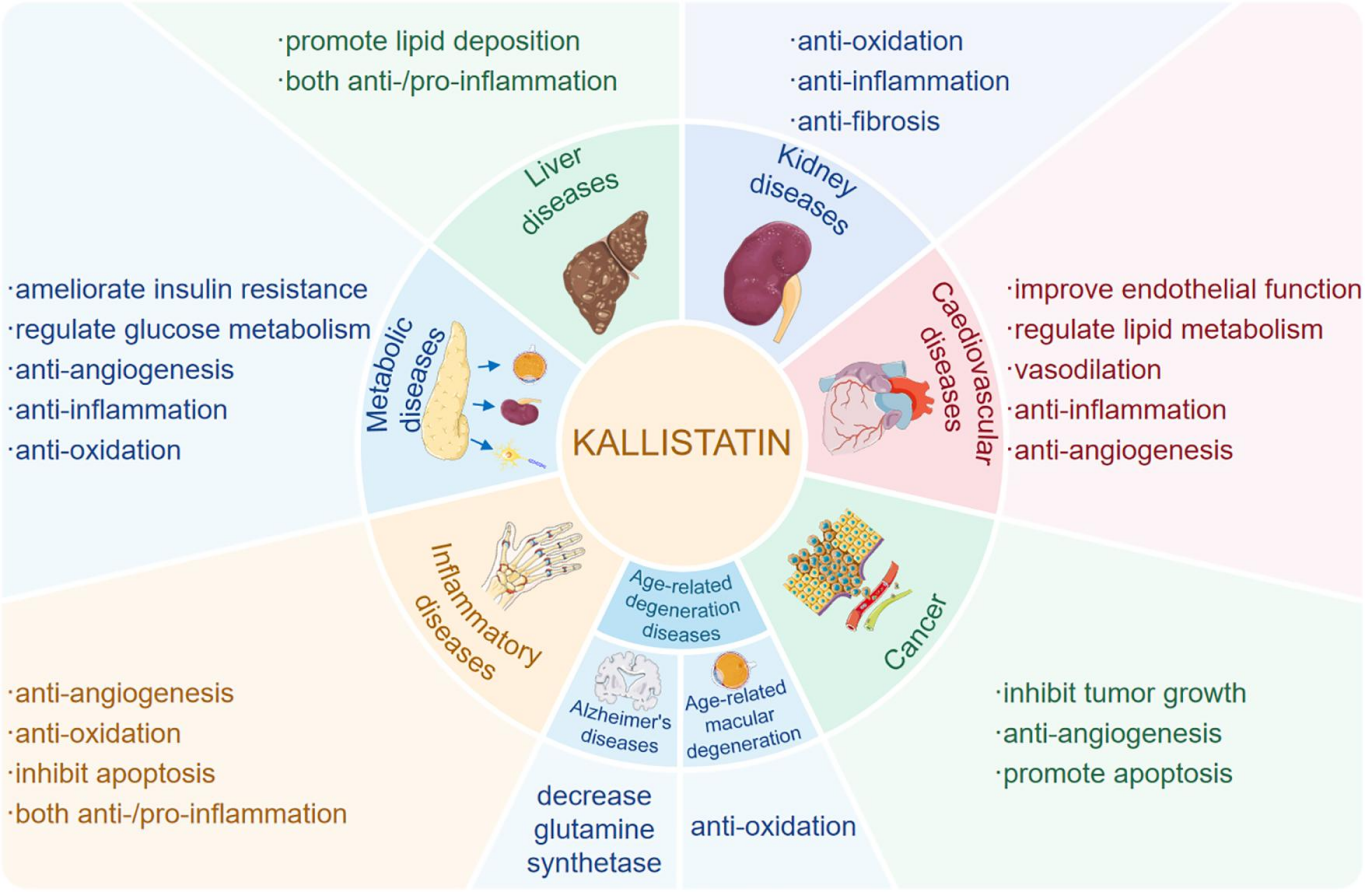
Kallistatin plays a multifaceted role in human diseases. Kallistatin plays multifaceted roles in human diseases, including cardiovascular diseases, hepatic diseases, renal diseases, immunometabolic diseases, age-related diseases and cancer though regulating various pathophysiological processes such as oxidative stress, inflammation, apoptosis and angiogenesis, and tumor growth.

Extensive evidence has established kallistatin as a critical regulator of angiogenesis, inflammation, apoptosis, oxidative stress and fibrosis in various human diseases (Figure 1). Importantly, its functional effects are context-dependent, exhibiting double-edged roles across different physiological and pathological settings. For example, kallistatin may promote survival and repair in cardiovascular system, while it induces apoptosis and inhibits angiogenesis in tumor microenvironments (6). The mechanistic basis of this duality resides in its unique structure, comprising an active site and a heparin-binding domain, and its interactions with a spectrum of specific receptors. Through selective engagement of these receptor partners, kallistatin modulates key signaling pathways, such as Wnt/*β*-catenin, NF-*κ*B, and SIRT1/eNOS, thereby eliciting context-specific cellular responses.

This review systematically summarizes the latest progress about the structure and signaling pathway, and pathophysiological functions of kallistatin with an emphasis on its diagnostic and therapeutic potential in human diseases. We focus on: (1) Systematically deciphering the mechanism underlying its receptor-mediated signal transduction, and delving into the explanation of its tissue-dependent functions as well as double-edged roles; (2) Integrating fragmented discoveries across cardiovascular, metabolic, liver, kidney, inflammatory and tumor diseases to form a unified understanding of kallistatin as a systemic modulator; (3) Assessing the challenges and opportunities of translational research, offering a forward-looking analysis on harnessing its duality for developing targeted therapeutic strategies. By integrating fragmented discoveries of kallistatin in different diseases, we aim to provide retrospective overview and prospective perspectives to the future mechanistic exploration and therapeutic development.

## 2 The structure and receptor signaling of kallistatin

**Figure 2:**
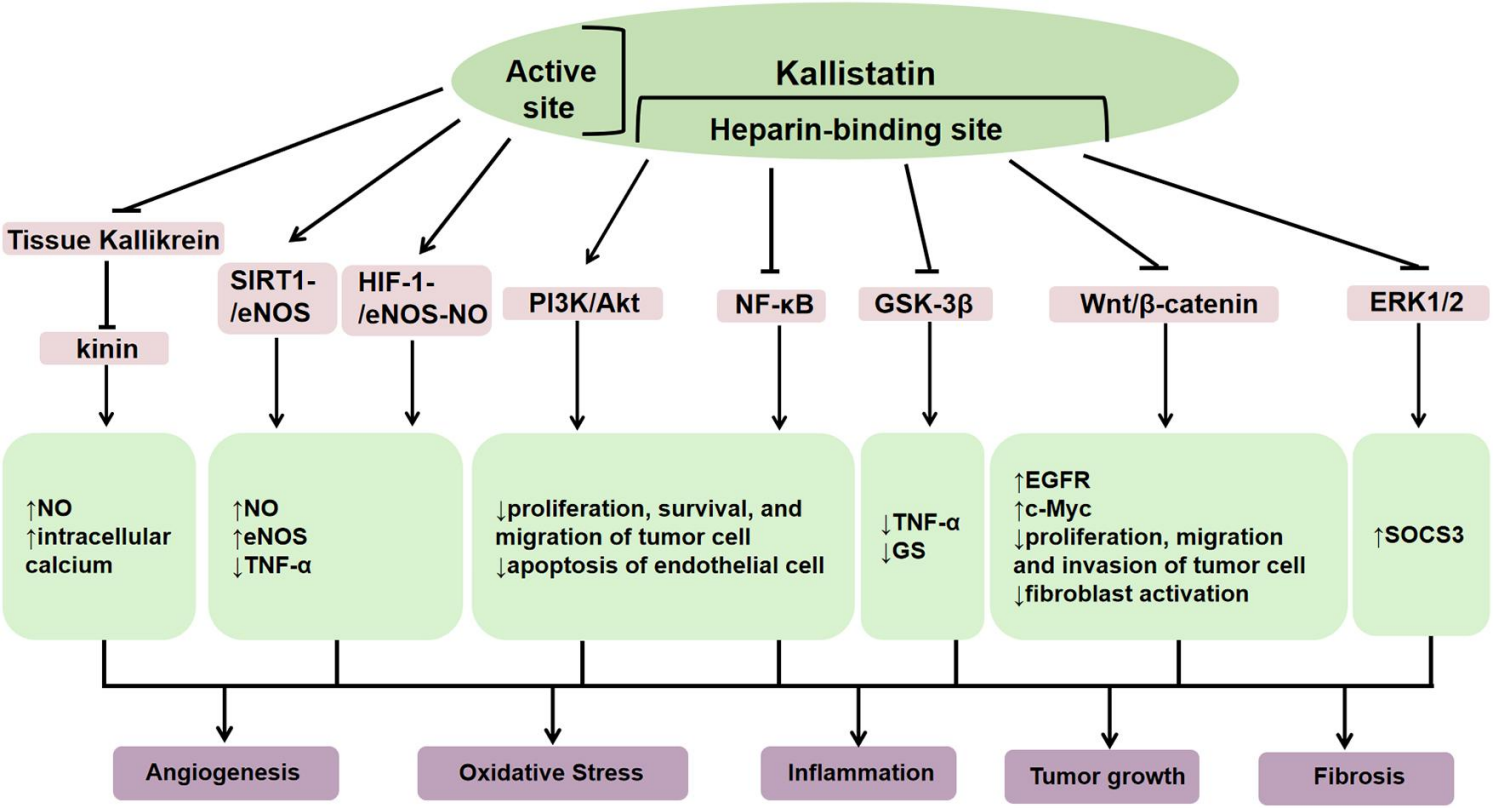
The structure of kallistatin and its functions. Kallistatin exerts diverse biological functions in the body through its heparin binding domain and active site. As a kallikrein binding protein, it can negatively regulate the kallikrein-kinin system (KKS) by directly inhibiting tissue kallikrein activity, thereby reducing bradykinin generation. Through this mechanism, kallistatin exerts protective functions in cardiovascular system, including vasodilation and anti-inflammatory, anti-apoptotic, and anti-fibrotic actions. Moreover, it can inhibit the migration and invasion of cancer cells through the supression activity on tissue kallikrein. Its heparin binding domain can inhibit oxidative stress, promote angiogenesis and vascular repair by stimulating the phosphatidylinositol 3-kinase/protein kinase B (PI3K/Akt) signaling pathway. It can also inhibit angiogenesis, inflammation, fibrosis, and tumor growth by suppressing glycogen synthase kinase 3*β* (GSK-3β), wnt/*β*-catenin, extracellular regulated protein kinases1/2 (ERK1/2), and nuclear factor kappa-B (NF-*κ*B). Trough binding to its active site, kallistatin mainly promotes the silent information moderating factor 1-endothelial nitric oxide synthase (SIRT1-eNOS) and hypoxia-inducible factor-1-endothelial nitric oxide synthase-nitric oxide (HIF-1-eNOS-NO) signaling pathways, inhibits inflammatory responses, and slows down vascular failure.

The human kallistatin gene (*SERPINA4*) is located on chromosome 14q31-32.1, and the rat *Serpina4* is located on chromosome 6. The mouse *Serpina4* is a pseudogene and *Serpina3*c is considered to be homolog of human *SERPINA4* (7). The translated amino acid sequence of human kallistatin shares 44%–46% homology with protein C inhibitor, *α*1-antitrypsin, *α*1-antichymotrypsin and rat kallikrein-binding protein (8). The secondary structure of kallistatin consists of three *β*-sheets and eight *α*-helices (9). Functionally, its core structure consists of an active site and a heparin-binding domain respectively (10), which is inseparable from biological functions (Figure 2). As a kallikerin binding protein, kallistatin is a major endogenous inhibitor that negatively regulates the kallikrein-kinin system (KKS) by directly inhibiting tissue kallikrein activity. Through its active site, it covalently binds to tissue kallikrein, which inhibits the cleavage of kininogen into vasoactive kinin peptides and thereby participates in blood pressure regulation, endothelial cell proliferation, and vascular repair (11). Additionally, kallistatin may antagonize endothelial nitric oxide synthase (eNOS), sirtuin 1 (SIRT1) and microRNA (miRNAs)-mediated inflammation, oxidative stress and tumor growth via its active site (12). Through the heparin-binding domain, kallistatin binds to cell surface heparan sulfate proteoglycans. Heparin, as a key glycosaminoglycan, can bind to kallistatin, and eliminate kallistatin's inhibitory effect on human tissue kallikrein while conferring anti-angiogenic capabilities and enhancing its inhibitory effects on other proteases, such as chymotrypsin (10). Its heparin binding domain enables kallistatin to regulate several signaling pathways, such as phosphatidylinositol 3-kinase/protein kinase B (PI3K/Akt), Wnt/*β*-catenin, extracellular regulated protein kinases1/2 (ERK1/2), and nuclear factor kappa-B (NF-*κ*B), which involved in angiogenesis, oxidative stress, apoptosis, inflammation, fibrosis, and tumor growth, and thereby significantly impacting human disease pathogenesis (13). The pleiotropic functions of kallistatin is mirrored by Klotho, which also exerts anti-inflammatory and anti-fibrotic effects across various organs by modulating TGF-*β*, NF-*κ*B, and MAPK pathways (12).

**Figure 3:**
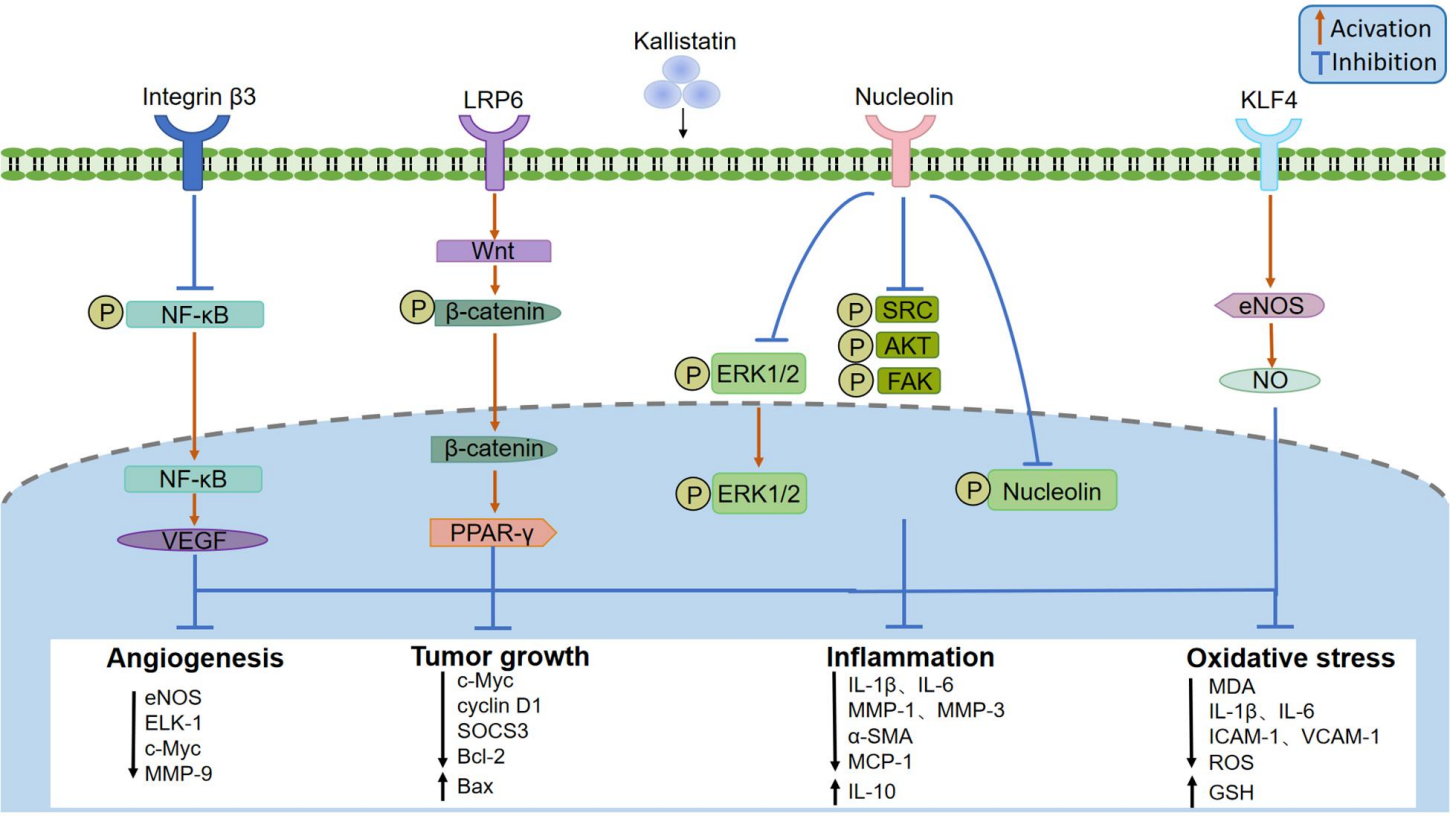
Kallistatin binding receptors and signaling pathway. Kallistatin signals through several receptors, including integrin *β*3, lipoprotein receptor-related protein 6 (LRP6), nucleolin, and Krüppel-like factor 4 (KLF4). Kallistatin can inhibit angiogenesis and tumor development by suppressing nuclear factor-*κ*B (NF-*κ*B), thereby inhibiting angiogenesis and vascular permeability mediated by vascular endothelial growth factor (VEGF), or by activating the wnt/*β*-catenin pathway through LPR6 and acting on peroxisome proliferator-activated receptor *γ* (PARR-*γ*), or by inhibiting the phosphorylation of tyrosine kinase (SRC), focal adhesion kinase (FAK), AKT, extracellular regulated protein kinases1 (ERK1) and extracellular regulated protein kinases 2 (ERK2) through nucleophosmin. After binding to KLF4, kallistatin can stimulate eNOS expression and NO production, playing an anti-inflammatory and anti-oxidative stress role.

Recently, several kallistatin binding proteins have been identified, including integrin *β*3, lipoprotein receptor-related protein 6 (LRP6), nucleolin, and Krüppel-like factor 4 (KLF4). By signaling through specific receptors, it exerts diverse biological functions (Figure 3). Kallistatin can directly bind integrin *β*3 on the cell surface of cultured small cell lung cancer cell line NCI-H446, and recombinant human kallistatin inhibits tumor cell growth, migration, invasion, and angiogenesis (14). Mechanistically, kalllistatin suppresses nuclear factor-*κ*B (NF-*κ*B) activity, leading to downregulation of vascular endothelial growth factor (VEGF) expression, a key factor in mediating angiogenesis. This subsequently blocks the phosphorylation of protein kinase B (AKT) and extracellular signal-regulated kinase (ERK) (15–17). As an essential Wnt/*β*-catenin pathway co-receptor, LRP6 enables to activate canonical Wnt signaling (18). The combination of kallistatin with LRP6 blocks the activation of Wnt/*β*-catenin signaling pathway and thus inhibits angiogenesis, inflammtion and the growth of cancer cell in cultured human breast cancer cells (19–21). Nucleolin is a common nucleolar protein that is highly expressed in the cytoplasm and nucleus of tumor cells (22). Once bound to nucleolin, kallistatin is internalized and transported into nucleus via nucleolin, where it inhibits nucleolin phosphorylation and its downstream activation of kinases SRC, FAK, AKT, and ERK1/2 to inhibit endothelial cell growth, tumor cell proliferation and angiogenesis (23). KLF4 is a zinc-finger transcription factor and may serve as a kallistatin binding protein. By co-immunoprecipitation studies in endothelial cells overexpressing KLF4, the interation of kallistatin and KLF4 was increased, and knockdown of KLF4 abolished the anti-inflammatory effect of kallistatin induced by tumor necrosis factor *α* (TNF-α) (24). The mechanisms of its anti-inflammatory and anti-oxidative stress effect are likely related to increase of NO production via eNOS in endothelial cells (25, 26). However, strong evidence is needed to determine whether kallistatin can directly bind to and regulate the activity of KLF4.

## 3 Role of kallistatin in human diseases

Recent studies have established kallistatin as a critical regulator in diverse pathophysiological processes. Kallistatin levels in circulating and tissues are closely associated with the development and progression of various human diseases (Table 1), highlighting its potential as both a diagnostic biomarker and therapeutic target. Notably, kallistatin has been proved to display a double-edged function in angiogenesis, apoptosis, and oxidative stress (27). For instance, it exerts pro-angiogenic, anti-apoptotic, and anti-oxidative effects in cardiovascular diseases, showing great therapeutic potential. Conversely, in cancers, kallistatin exerts anti-angiogenic and pro-apoptotic function, inhibiting tumor proliferation, growth, and metastasis. Subsequent sections will systematically delineate the expression properties, specific effects, signaling pathways, and molecular mechanisms in disease contexts.

**Table 1:** The expression levels of kallistatin in human diseases.

Diseases			Levels of kallistatin (Diseases vs. Controls)
Cardiovascular Diseases	Preeclampsia		Serum ↓ (33)
	Pulmonary Arterial Hypertension		Serum ↓ (34)
	Atherosclerosis		Plasma ↓ (38)
	Heart Failure		Plasma ↓ (44)
Liver Diseases	Non-obese patients with Non-Alcoholic Fatty Liver Disease (NAFLD)		Serum ↑ (20)
	Obese patients with NAFLD		Liver ↓ (47)
	Liver Fibrosis		Serum ↓ (49)
	Liver Cirrhosis		Serum ↓ (50)
	Alcohol-Associated Hepatitis		Plasma ↓ (48)
	Alcoholic Liver Cirrhosis		Serum ↓ (51)
Kidney diseases	Chronic Kidney Disease		Kidney ↓ (54)
Metabolic Diseases	Diabetes	Type 1 Diabetes	Serum ↑ (62, 66), Plasma ↑ (65)
		Type 2 Diabetes	Subcutaneous white adipose tissue (sWAT) mRNA ↓ (67)
	Diabetic complications	Diabetic Retinopathy	Vitreous humor ↓ (70, 71)
		Diabetic Nephropathy	Serum ↑ (73)
		Diabetic Sensorimotor Neuropathy	Serum ↓ (79)
	Polycystic Ovary Syndrome		Serum ↓ (83)
Inflammatory Diseases	Rheumatoid Arthritis		Plasma, Joint fluid ↑ (84)
	Autoimmune Uveitis		Plasma ↑ (92)
	Chronic Rhinosinusitis		Nasal polyp tissue ↑ (91)
	Community-Acquired Pneumonia		Serum ↓ (88)
	Tubo-Ovarian Abscess		Serum ↓ (94)
	Sepsis		Serum ↓ (96), Plasma ↓ (5) (97)
Cancer	High-grade Serous Ovarian Cancer		High-grade serous ovarian cancer tissue ↓ (111)
	Colorectal Cancer		Colorectal cancer tissue ↓ (98)
	Gastric Cancer		Gastric cancer tissue ↓, Metastatic lymph nodes ↓, Plasma ↓ (103)
Age-related degeneration diseases	Age-related Macular Degeneration		Plasma ↓ (116)
Others	Placenta Accreta Spectrum		Plasma ↑ (118)
	Preterm Labor		Plasma ↓ (119) Amniotic fluid ↑ (120) Cervicovaginal fluid ↑ (121)

### 3.1 Cardiovascular diseases

#### 3.1.1 Hypertension

Kallistatin is widely distributed in endothelial cells and smooth muscle cells of blood vessels implicating its potential role in regulating vascular function (28). As an effective vasodilator, intravenous human kallistatin rapidly and transiently reduces arterial blood pressure in rats, which may function directly through a vascular smooth muscle mechanism independent of an endothelial bradykinin receptor (29). In addition, circulating levels of rat KBP, the analogue of human kallistatin, are significantly reduced in spontaneously hypertensive rats compared to normotensive controls (30), while transgenic rat-KBP overexpression lowers blood pressure in mice compared to controls (31). Interestingly, a latest study using UK Biobank data demonstrated rs1014754, an identified hypertension-associated single nucleotide polymorphism (SNP), was positively correlated with kallistatin in the Fenland cohort. These findings highlight kallistatin's counterregulatory role with rennin in blood pressure modulation, positioning it as a promising therapeutic target for hypertension (32).

Kallistatin may also play a role in secondary hypertension, as evidence by lower serum kallistatin levels in women with preeclampsia (33) and patients with pulmonary arterial hypertension (PAH) (34). Meanwhile, serum kallistatin levels were negative correlation with systolic and diastolic blood pressure, and creatinine in women with preeclampsia (33). Mechanic studies show that the active site of kallistatin can induce endothelial nitric oxide synthase (eNOS) activation and nitric oxide (NO) production in endothelial cells, ultimately driving vasodilation and blood pressure reduction (2, 13, 35). Therefore, these combined findings indicate that kallistatin may serve as a new vasodilator and an endogenous blood pressure-lowering agent.

#### 3.1.2 Atherosclerosis

Atherosclerosis is the primary cause of cardiovascular diseases, including coronary artery disease (CAD, also known as coronary heart disease), peripheral artery disease, and stroke. Emerging evidence indicates that kallistatin is able to antagonize the development of atherosclerosis, highlighting its therapeutic potential for atherosclerosis-related cardiovascular diseases (9, 36). Studies have demonstrated that serum kallistatin levels are decreased in CAD patients and negatively correlated with the Gensini score and triglyceride levels, and disease severity (37, 38). In addition, serum kallistatin concentrations show a positive correlation with high-density lipoprotein cholesterol (HDL-C) levels and an inverse association with low-density lipoprotein cholesterol (LDL-C) concentrations (39, 40). Notably, HDL-C exerts atheroprotective effects whereas LDL-C promotes proatherogensis (41). These studies highlight circulating kallistatin's promise as a biomarker for atherosclerosis.

Recent studies have shown that the protective mechanisms of kallistatin against atherosclerosis mainly include improving endothelial dysfunction, anti-vascular inflammation, and regulating lipid metabolism (9). Kallistatin promotes eNOS expression, thereby stimulating NO production, which maintains the normal morphology of blood vessels. Human kallistatin gene transfer prevents carotid atherosclerotic plaque formation, reduces macrophage deposition, tumor necrosis factor-α (TNF-α) expression and oxidative stress in apoE^–/–^ mice through upregulating SIRT1/eNOS pathway (38). Moreover, kallistatin inhibits apoptosis in human umbilical vein endothelial cells (HUVECs) via the phosphatidylinositol-3-kinase (PI3K)/Akt/eNOS signaling pathway (25). However, further research is required to elucidate its pathophysiological mechanisms and clinical application potential.

#### 3.1.3 Heart failure

Heart failure (HF) is a prevalent cardiovascular disease characterized by high morbidity, mortality, and poor prognosis (42, 43). Decreased plasma kallistatin levels have been identified as both a risk factor and a prognostic marker for HF readmission (44). Supporting this clinical observation, gene deficiency of *Serpina3c* in mice, a homolog of human *SERPINA4*, aggravates myocardial fibrosis and promotes cardiac fibroblast proliferation after myocardial infarction (MI) via Nr4a1/ENO1/glycolysis pathway (44). Conversely, kallistatin gene therapy in MI animal models inhibits cardiac injury and remodeling, as well as hypertrophy by inhibiting oxidative stress and modulating the SIRT1/peroxisome proliferators-activated receptor *α* (PPAR*α*) pathway (45). Additionally, kallistatin treatment effectively improves the cardiac function, and attenuates inflammation and apoptosis of myocardial tissue by promoting SIRT1 in HF rats (46). These studies indicate kallistatin as a potential target for prevention and treatment of HF.

### 3.2 Liver diseases

Kallistatin is mainly synthesized and secreted by the liver, suggesting its critical roles in liver diseases, including hepatic steatosis, non-alcoholic steatohepatitis (NASH), and cirrhosis. Current evidence suggests kallistatin may exhibit dual roles as both a pathogenic mediator and a protective modulator in the liver. Hyperlipidemia is a significant risk factor for hepatic steatosis and nonalcoholic fatty liver disease (NAFLD). Fang et al. reported significantly elevated serum kallistatin levels in hyperlipidemic subjects and NAFLD patients compared to normal people, which may be partially attributed to the antagonistic effect of elevated free fatty acids on the inhibitory action of thyroid hormone T3 on kallistatin expression (20). Mechanistically, kallistatin overexpression induced hepatic steatosis and NAFLD in animal models by inhibiting comparative gene identification-58 (CGI-58) and lipotriglyceride lipase (ATGL) via the LRP6/G*α*s/PKA/GSK3*β* pathway. Furthermore, it promotes the nuclear translocation of NF-*κ*B p65, inducing TNF*α* transcription and thereby contributing to chronic liver inflammation (20). Contrastingly, Frühbeck et al. demonstrated significant reduced circulating kallistatin in human obesity, and *SERPINA4* gene expression levels were downregulated in the liver of obese patients with NAFLD (47). Kallistatin treatment protects human adipocytes against inflammation and oxidative stress by inhibiting TNF-α and inducing SIRT1 signaling (47). Plasma proteomic profiling in patients with alcohol-associated hepatitis (AH) revealed a significant downregulation of kallistatin compared to healthy controls, with kallistatin levels exhibiting strong association with disease severity (48). Moreover, serum kallistatin levels are significantly lower in patients with liver fibrosis (49, 50), cirrhosis (50) and alcoholic cirrhosis (51) compared to controls, and decrease further with disease progression (50, 51), indicating it as a useful and reliable diagnostic biomarker for hepatic health. In rodent models of carbon tetrachloride (CCl4)-induced liver injury, kallistatin treatment significantly alleviates oxidative stress, inflammation and fibrosis in the liver (52, 53). Collectively, these findings suggest that kallistatin may be helpful for diagnosing or monitoring disease progression of diverse chronic liver diseases. However, therapeutic targeting requires caution due to its context-dependent modulation of opposing functions: it can simultaneously drive pro-inflammatory/pro-fibrotic cascades while mediating anti-inflammatory/anti-fibrotic responses. Additionally, further investigations are needed to elucidate the specific molecular mechanisms underlying roles of kallistatin in cirrhosis.

### 3.3 Kidney diseases

Kallistatin has been demonstrated to protect against kidney diseases, including acute kidney injury (AKI) and chronic kidney disease (CKD) (13). Within the human kidney, kallistatin is predominantly localized to renal tubules, with highest abundance in the distal tubules and collecting ducts, followed by Henle's loops and proximal tubules (13, 54). Recent studies have indicated that kallistatin gene variants are associated with AKI in patients with septic shock. Specifically, patients carrying the rs2093266 SNP in *SERPINA4* have a significantly reduced risk of AKI (55, 56). The renal protective role of kallistatin in AKI is further confirmed in septic animal models. In mice with polymicrobial sepsis, kallistatin treatment attenuates lethality, reduces peritoneal bacterial counts, mitigates renal injury and inflammation (4, 57). More clinical and molecular studies are needed to figure out the role and molecular mechanisms of kallistatin in AKI. In CKD patients, renal kallistatin expression is reduced, exhibiting a positive correlation with estimated glomerular filtration rate (eGFR) and a negative correlation with serum creatinine (54). In a unilateral ureteral obstruction (UUO) mouse model, anti-kallistatin antibody exacerbates renal injury by promoting extracellular matrix (ECM) deposition and fibroblast activation via the Wnt/*β*-catenin signaling pathway, indicating its protective role in tubulointerstitial fibrosis (54). In addition, kallistatin levels are reduced in the kidneys under hypertension. Transgenic kallistatin overexpression ameliorates oxidative stress, inflammation, and fibrosis in hypertension-induced renal injury (31, 58–60). These studies suggest that kallistatin holds significant potential to serve as an important therapeutic target for kidney diseases.

### 3.4 Metabolic diseases

#### 3.4.1 Diabetes and its complications

##### 3.4.1.1 Diabetes

Kallistatin plays diverse roles in diabetes and its complications, due to its pleiotropic role in regulating angiogenesis, oxidative stress, inflammation, and fibrosis. Current clinical evidence demonstrates a complex dynamic in its circulating levels: while no significant difference exists between diabetic and non-diabetic patients (61), notable elevations occur in prediabetic individuals (62), type 1 diabetes (T1D) patients (63, 64), diabetes and diabetic foot ulcer patients (65), and obese patients with type 2 diabetes (T2D) (66). Conversely, kallistatin expression is significantly upregulated in diabetic tissues, such as subcutaneous adipose tissue (67) and corneas (68). Notably, as an endogenous Wnt signaling inhibitor, kallistatin administration improves hepatic insulin resistance, while delays corneal wound healing by inhibiting Wnt/*β*-catenin signaling (67, 68). Although these preliminary findings underscore kallistatin's dual effects in diabetes, more clinical studies are warranted to delineate its expression pattern and tissue-specific mechanisms.

##### 3.4.1.2 Diabetic retinopathy

Diabetic retinopathy (DR) is one of sever microvascular complications of diabetes, with chronic inflammation as the major pathological mechanism (69). Kallistatin levels in vitreous humor of patients with DR (70) and proliferative DR (71) are significantly reduced compared to nondiabetic controls. With further research, kallistatin has been proven to bind to LPR6 to inhibit diabetes-induced Wnt/*β*-catenin signaling pathway and attenuates inflammation and angiogenesis in DR (72).

##### 3.4.1.3 Diabetic nephropathy

Diabetic nephropathy (DN) is also a very common complication of diabetes, as characterized by proteinuria, reduced glomerular filtration, thickened glomerular basement membrane and renal fibrosis. Serum kallistatin levels are significantly elevated in T2D patients with DN compared to T2D patients without DN and normal controls (73). However, renal kallistatin expression is reduced in diabetic mice and inversely correlates with circulating levels. Renal kallistatin overexpression protects against DN by ameliorating oxidative stress, inflammation, and fibrosis, potentially through inhibiting AGE-RAGE axis and microRNA-34a signaling (74, 75). In contrast, increased kallistatin levels in serum may promote DN progression by inhibiting tissue kallikrein activity, thereby attenuating the protection of kallikrein-kinin system and inducing renin-angiotensin system (RAS) overactivation (76). These findings suggest the dual role of kallistatin in DN progression is probably mediate by its differential expression in circulation and tissues, and the underlying mechanism remains to be further investigated.

##### 3.4.1.4 Diabetic sensorimotor neuropathy

Diabetic sensorimotor neuropathy (DSPN), another microvascular complication, significantly impairs patient survival and quality of life. Its pathogenesis involves oxidative stress and endothelial dysfunction (72, 77). Alpha-lipoic acid (ALA), a mitochondrial cofactor with potent antioxidant properties, demonstrates therapeutic efficacy in DSPN (78). Following ALA therapy, serum level of kallistatin in patients with DSPN decreased significantly, suggesting its utility as a potential biomarker for monitoring treatment response (79). Given kallistatin's biological functions, ALA may alleviate DSPN symptoms through kallistatin-mediated antioxidant and vascular protective effects, though further studies are needed.

#### 3.4.2 Polycystic ovary syndrome

Polycystic ovary syndrome (PCOS), the most common endocrine-metabolic disorder in reproductive-aged women, presents diagnostic challenges due to frequent comorbid conditions (80–82). Current evidence suggests a potential association between serum kallistatin levels and PCOS pathogenesis. Notably, study has found significant lower serum kallistatin levels in PCOS patients compared to healthy controls (83), which suggests that kallistain may be used as a diagnostic biomarker for PCOS.

### 3.5 Inflammatory diseases

#### 3.5.1 Rheumatoid arthritis

Rheumatoid arthritis (RA) represents a chronic systemic autoimmune disorder, primarily driven by inflammation and autoimmune reactions. Recent studies demonstrated that circulating and joint fluid levels of kallistatin are significantly higher in RA patients compared to osteoarthritis (OA) patients (84). Further analysis found that elevated expression of kallistatin mainly localizes in fibroblast-like synoviocytes and mononuclear cells of synovial tissues from RA patients (84). In animal models, administration of kallistatin suppresses RA development (85, 86) and OA progression (87) through inhibiting angiogenesis, inflammation and apoptosis. Mechanistically, kallistatin via its heparin-binding domain, ameliorates synovial inflammation by blocking TNF-α and NF-*κ*B signaling activation, and inflammatory gene transcription (13). Therefore, these findings suggest that kallistatin holds promise as a therapeutic strategy for arthritis.

#### 3.5.2 Acute lung injury

Kallistatin has been shown to provide protection against pneumosepsis, acute respiratory distress syndrome (ARDS), and lung inflammation. Lin and colleagues showed that serum levels of kallistatin in 54 severe community-acquired pneumonia (CAP) patients admitted to intensive care unit were significantly lower than those in 17 healthy controls, indicating substantial consumption of kallistatin during disease process (88). Consistently, plasma kallistatin levels are markedly decreased in patients with septic shock or those developing acute respiratory distress syndrome (ARDS). Meanwile, lower kallistatin levels strongly correlate with higher mortality and greater disease severity in CAP and COPD patients (13, 88, 89). Critically, animal models have validated kallistatin's protective role in sepsis-associated acute lung injury through suppressing intracellular reactive oxygen species (ROS) generation and NF-*κ*B activation, and stimulating tyrosine-kinase-protein kinase C-extracellular signal-regulated kinase (ERK) signaling, thereby alleviating inflammatory responses (4, 90). Thus, these findings highlight kallstatin as a novel predictive biomarker and therapeutic target for acute lung injury.

#### 3.5.3 Chronic rhinosinusitis

Chronic rhinosinusitis with nasal polyps (CRSwNP) is a complex inflammatory airway disorder airway. Recent studies demonstrate elevated kallistatin expression in nasal polyp tissues of CRSwNP patients compared to normal nasal mucosa, with levels positively correlating with inflammatory cytokines (91). Notably, kallistatin levels are further increased in uncontrolled, partially controlled, and asthma-associated CRSwNP patients, suggesting its potential as a biomarker for disease severity and prognosis. Moreover, kallistatin overexpression in mice promotes Th2-type inflammatory responses by inducing IL-4 expression and CD4^+^ T cell secretion, thereby contributing to pathogenesis of CRSwNP (91).

#### 3.5.4 Autoimmune uveitis

Autoimmune uveitis, a severe ocular inflammatory disease frequently leads to visual impairment or blindness. Compared with non-uveitis controls, plasma kallistatin levels are significantly upregulated in patients with Vogt-Koyanagi-Harada (VKH) disease, a type of non-infectious uveitis (92). Paradoxically, in experimental autoimmune uveitis models, transgenic kallistatin overexpression in mice exacerbates uveitis symptoms through promoting Th17 cell-mediated inflammatory responses (92), whereas suppresses disease severity through inhibiting T cell activation (93). These indicate that kallistatin may play a double edge effect with both anti-inflammatory and pro-inflammatory properties in autoimmune uveitis. This dual role of kallistatin has also been reported in DN, potentially attributable to its different levels in tissues and system. Thus, local ocular delivery of kallistatin represents a promising strategy to maximize therapeutic efficacy while minimizing systemic consequences. Further investigation into its compartment-specific molecular mechanisms is warranted to develop novel treatments for autoimmune uveitis.

#### 3.5.5 Tubo-ovarian abscess

Tubo-ovarian abscess (TOA) is a rare but serious manifestation of pelvic inflammatory disease, with significant life-threatening risks. Analysis of serum kallistatin levels revealed significant reductions in 30 hospitalized TOA patients compared to 30 control patients undergoing elective surgery, suggesting its diagnostic potential for TOA. However, the study was limited by its small sample size, and larger-scale studies are needed in the future to further validate the effectiveness and accuracy of serum kallistatin as a diagnostic marker for TOA (94).

#### 3.5.6 Sepsis

Sepsis, defined as a a systemic inflammatory response syndrome (SIRS), is triggered by microbial infection or bacterial products, such as lipopolysaccharide (LPS). Kallistatin levels can be consumed during sepsis in both clinical patients and animal models, which may indicate a protective role to prevent blood pressure lowering (5, 95). A retrospective study demonstrated that decreased serum kallistatin level independently predicts 28-day mortality in septic shock patients, a more severe sepsis condition (96). Consistently, plasma kallistatin levels on ICU admission day 1 are progressively lower in septic shock patients compared with sepsis patients, and non-survivors compared with survivors, indicating that a decrease plasma kallistatin concentration reflects increased severity and poorer outcome of disease (97). In mouse models of polymicrobial sepsis and endotoxemia, delayed administration of recombinant human kallistatin significantly increased the survival rate of mice and reduced key pro-inflammatory mediators (TNF-α, IL-6, and HMGB1) in the serum (4). These findings suggest that kallistatin holds potential as a diagnostic biomarker for sepsis, prognostic indicator for septic shock, and promising therapeutic target.

### 3.6 Cancer

#### 3.6.1 Colorectal cancer

Emerging evidence supports kallistatin as a candidate early prognostic biomarker and therapeutic target for colorectal cancer (CRC). Kallistatin expression is significantly downregulated in CRC tissues compared to normal colorectal mucosa. Moreover, patients lacking kallistatin expression exhibit higher recurrence rates than those with weak or positive expression (98). In both *in vivo* and *in vitro* models, kallistatin overexpression markedly inhibits angiogenesis, and proliferation, migration, and invasion of colorectal cancer cells. Mechanistically, kallistatin exerts tumor-protective effects through inhibiting the Wnt/*β*-catenin signaling pathway by interaction with LRP6 and the PPAR*γ*/Fas/FasL signaling axis (99–101).

#### 3.6.2 Gastric cancer

Gastric cancer (GC), ranked fifth globally in both incidence and mortality among all cancers, predominantly metastasizes via the lymphatic system (102). Kallistatin expression is significantly downregulated in GC tissues, metastatic lymph nodes, and plasma of GC patients (103). Serum kallistatin is specific for early diagnosis of advanced GC, independent of H. pylori infection, a major GC risk factor (103, 104). Further functional evidence using recombinant kallistatin or adenovirus-mediated kallistatin overexpression in human gastric cancer cells confirms the direct anti-lymphangiogenic effects of kallistatin through binding to LRP6 and inhibiting the IKK/I*κ*B/NF-*κ*B signaling, consequently downregulating VEGF-C expression and secretion (103, 105, 106).

#### 3.6.3 Lung cancer

Studies have demonstrated that serum kallistatin, combined with PON1 and age in a logistic regression model, improves differential diagnosis of lung cancer vs. non-cancerous lung diseases. Kallistatin levels are significantly downregulated in the serum of lung-cancer patients, and both gene-delivery and recombinant-protein interventions of kallistatin establish its capacity to suppress angiogenesis, inflammation and tumor metastasis (107). Functionally, kallistatin administration inhibits the proliferation, survival, and migration of lung cancer cells through binding integrin *β*3 to suppress PI3K/AKT signaling casdase (14, 108, 109). However, more clinical studies are warranted to elucidate the underlying molecular mechanisms.

#### 3.6.4 Ovarian cancer

High-grade serous ovarian cancer (HGSOC) is a predominant subtype of ovarian cancer with poor prognosis and high mortality (110). Studies have shown that kallistatin is significantly decreased in HGSOC patients compared to normal fallopian tube tissue, and patients with higher kallistatin expression have longer survival times. Additionally, kallistatin administration inhibits migration and invasion, and promotes apoptosis of ovarian cancer cells (111). These findings suggest that kallistatin may serve as a novel prognostic biomarker and a potential therapeutic target for HGSOC.

#### 3.6.5 Cervical cancer

In both cultured HeLa/SiHa cells and nude mice with HeLa xenograft tumors, recombinant human kallistatin intervention could inhibit the viability of cervical cancer cells and promote apoptosis. Kallistatin blocks the NF-*κ*B signaling pathway by inhibiting I*κ*B*α* degradation and p65 phosphorylation, thereby inhibiting proliferation, migration, and invasion of cervical cancer (112).

#### 3.6.6 Breast cancer

Kallistatin may exert potent anti-tumor effects in breast cancer through various signaling pathway. Kallistatin inhibits breast cancer cell proliferation, migration, and invasion by binding LRP6 to block Wnt/*β*-catenin signaling (113), with its heparin-binding site antagonizing Wnt3a-induced proliferation (19). Meanwile, it suppresses TNF-α-activated NF-*κ*B signaling to impede cell migration and inhibit tumor angiogenesis (17). Through its active site, kallistatin downregulates oncogenic miR-21 and miR-203 while upregulating tumor-suppressive miR-34a and p53, thereby inducing apoptosis and autophagy of breast cancer cells (19). Notably, it should pay attention that the heparin-binding site of kallistatin inhibits endothelial cell apoptosis, whereas its active site promotes breast cancer cell apoptosis (27). This contradictory effects of kallsitatin on apoptosis suggest kallistatin's domain-specific targeting of receptor signaling pathways may be a promising therapeutic strategy for precision oncology in breast cancer.

### 3.7 Age-related degeneration diseases

Kallistatin has been proven to be involved in the progression of Alzheimer's disease (AD) (114). Though analysis of the dataset (GSE48350) indicated upregulated kallistatin mRNA expression in the hippocampus of patients with AD, direct evidence regarding kallistatin expression in plasma or neural tissues of AD patients remains scarce. Animal studies demonstrated that kallistatin transgenic mice exhibit reduced cognitive function and impaired glutamate homeostasis. Mechanistically, kallistatin induces acetylation and degradation of glutamine synthetase (GS) via the GSK-3β/SIRT1 pathway. The reduction of GS compromises glutamate-glutamine cycle homeostasis, representing an early pathogenic mechanism in cognitive and memory impairment. Age-related macular degeneration (AMD) is a leading cause of irreversible vision loss in older adults and is still lack of effective treatments. It is reported that while kallistatin may inhibit AMD. Kallistatin levels are reduced in the serum of patients with AMD and in the retina of sodium iodate-induced AMD rats (115, 116). Notably, serum kallistatin levels below a certain threshold indicate increasing risk of AMD (116). Kallistatin is involved in the epithelial-mesenchymal transition of retinal pigment epithelial cells during AMD pathogenesis. Kallistatin knockout rats show increased EMT and reactive oxygen species generation, while KAL overexpression inhibits these pathological effects. Kallistatin suppresses oxidative stress-induced EMT by downregulating the transcription factor Snail, suggesting its potential as a therapeutic target for dry AMD (115).

### 3.8 Other diseases

Kallistatin may play a role in many other diseases, such as inflammatory bowel disease, preterm labor, and placenta accreta spectrum (5, 117–121). For example, kallistatin levels in the plasma and intestine are significantly decreased in ulcerative colitis (UC) and Crohn's disease (CD) patients compared with controls (5, 122). However, more studies are needed to uncover the specific mechanisms of kallistatin in these diseases.

## 4 Conclusions

Initially identified as a kallikrein-binding protein, kallistatin is now demonstrated to play multifaceted roles in pathological and physiological conditions. Kallistatin is involved in the pathophysiology process of various human diseases, including cardiovascular diseases, hepatic diseases, renal diseases, immunometabolic diseases, and cancer. Signaling through its two functional binding sites and receptors, kallistatin regulates angiogenesis, oxidative stress, inflammation and fibrosis in a variety of tissues and organs. In this review, we reviewed the structure, receptors, signaling pathway and context-dependent role of kallistatin in human diseases.

The expression levels of kallistatin in plasma or tissues are closely related to organ function in different diseases, rendering it a potential biomarker for early diagnosis and progression monitoring. Its expression changes in the plasma or tissues are closely related to organ functional indicators in different diseases. However, in some diseases, such as liver diseases, the expression levels of kallistatin in the tissues and serum are inverse, potentially exerting opposing roles in the progression of the disease. High levels of kallistatin in the liver are associated with the progression of NAFLD, but decreased serum levels in patients with cirrhosis and liver fibrosis may indicate severity of disease. Given that plasma kallistatin is primarily synthesized in the liver, it is not surprising that plasma levels are low in liver cirrhosis and fibrosis. Furthermore, tissue-specific mechanisms, including local hormonal signaling, neural control, and metabolic product feedback can affect the synthesis, release and utilization of kallistatin. For example, elevated free fatty acid levels are likely to reverse the downregulation of kallistatin induced by thyroid hormone T3 (20). While in chronic kidney disease, proteinuria may contribute to hypokalistatinemia by impairing renal tubular reabsorption of kallistatin (54). Other acute conditions (e.g., acute pancreatitis and sepsis), decreased kallistatin levels might reflect increased consumption, driven by its binding to released tissue kallikrein or glycosaminoglycans (such as kallistatin, plasminogen activator inhibitor-1, and *α*1-antitrypsin) (10).

The roles of kallistatin across tissues are related to various factors, and its function is strictly governed by a complex network of multi-domain, multi-receptors, and multi-pathway interactions. Through binding to its heparin-binding site, active site, or specific receptors, kallistatin exerts dual regulatory effects on angiogenesis, oxidative stress, apoptosis, and inflammation. In tumorigenesis, kallistatin primarily acts as a suppressor by binding to LRP6 to inhibit the oncogenic Wnt/*β*-catenin pathway and by suppressing PI3K/Akt and NF-*κ*B signaling via its heparin-binding domain, thereby inducing apoptosis and blocking angiogenesis. Paradoxically, within the cardiovascular system, kallistatin utilizes the same structural domains to exert protective effects. Specifically, its active site activates the PI3K/Akt/eNOS signaling axis, enhancing endothelial cell survival, vasodilation, and vascular repair (27). Additionally, kallistatin's inhibition of NF-*κ*B signaling and TNF*α*-mediated inflammation through its heparin-binding domain confers tissue-specific therapeutic benefits. In tumors, this leads to reduced pro-tumorigenic inflammation and VEGF expression, whereas in non-malignant tissues, it mitigates endothelial dysfunction and inflammatory damage (6). This profound functional duality, while therapeutically appealing, presents significant translational challenges. First, organ-specific delivery strategies, such as ligand-functionalized nanoparticles or gene therapies driven by tissue-specific promoters are required to maximize efficacy and minimize off-target effects. Moreover, defining the therapeutic window requires critical trade-offs based on disease staging and patient comorbidities. For example, in early-stage cancers, kallistatin's anti-angiogenic and pro-apoptotic effects are desirable. However, in advanced disease or in patients with pre-existing cardiovascular conditions, strategies to temporally and spatially constrain its activity are essential to avoid impairing vascular repair. Similarly, in chronic diseases like hypertension, long-term kallistatin therapy must be balanced against its potential impact on wound healing. Finally, combination therapies involving kallistatin and conventional treatment modalities warrant further investigation to enhance therapeutic outcomes. For example, co-administration with chemotherapeutic agents may yield synergistic antitumor effects, but optimal timing and sequencing are essential to mitigate chemotherapy-induced vascular toxicity. Such studies will be essential to fully harness the potential of kallistatin in human diseases and the development of appealing and novel therapeutic approaches.
